# Association between the geriatric nutritional risk index and 28-day mortality in critically ill sepsis-associated pneumonia patients: retrospective study based on two cohorts

**DOI:** 10.3389/fnut.2025.1698973

**Published:** 2026-01-26

**Authors:** Dandan Shao, Linyu He, Dayang Zhong, Kun Li, Xianming Zhang

**Affiliations:** 1 Department of Respiratory and Critical Care Medicine, Affiliated Hospital of Guizhou Medical University, Guiyang, China; 2 School of Clinical Medicine, Guizhou Medical University, Guiyang, China; 3 Department of Respiratory and Critical Care Medicine, The Second People's Hospital of Guiyang, Guiyang, China

**Keywords:** dangerous stratification, geriatric nutritional risk index, intensive care, pneumonia, sepsis

## Abstract

**Background:**

In recent years, the identification of reliable prognostic indicators for critically ill patients has become increasingly crucial. The Geriatric Nutritional Risk Index (GNRI), a simple and objective tool for assessing malnutrition risk, has demonstrated significant prognostic value across various disease conditions. This study aims to investigate and validate the clinical utility of GNRI in predicting 28-day mortality among critically ill patients with sepsis-associated pneumonia.

**Material and method:**

We conducted a retrospective analysis using two distinct cohorts. Critically ill elderly people with sepsis-associated pneumonia were included. The derivation cohort consisted of critically ill patients with sepsis-associated pneumonia extracted from the MIMIC-IV database, while the validation cohort comprised consecutively enrolled patients meeting identical criteria from Jinyang Hospital Affiliated to Guizhou Medical University between March 2023 and March 2025. Key baseline variables including demographics, comorbidities, and severity scores were analyzed. We employed restricted cubic spline regression, multivariable logistic and Cox regression, and Kaplan-Meier analysis to assess associations between GNRI and mortality. Using LASSO regression for variable selection coupled with multivariable Cox proportional hazards modeling, we developed a prognostic nomogram across three distinct risk strata. Model discrimination was evaluated using time-dependent receiver operating characteristic (ROC) analysis, with predictive performance quantified by the area under the curve (AUC).

**Result:**

The final analysis included 2,230 critically ill patients with sepsis-associated pneumonia, with observed 28-day ICU and in-hospital mortality rates of 26.64% and 26.59%, respectively. In fully adjusted models, both continuous GNRI and categorical GNRI showed significant associations with 28-day ICU mortality across cohorts. The hazard ratios were 0.99 (95% CI: 0.98–1.00) for continuous GNRI; 0.69 (0.51–0.93) for moderate vs. high nutritional risk; and 0.41 (0.25–0.69) for no vs. high nutritional risk. Similar associations were observed for 28-day in-hospital mortality (no vs. high nutritional risk: HR: 0.59, 95% CI: 0.36–0.98). Restricted cubic spline analysis revealed a nonlinear relationship between continuous GNRI and both 28-day ICU and in-hospital mortality. When combined with conventional critical illness severity scores, GNRI provided incremental predictive value for 28-day mortality. ROC curve analysis demonstrated that our risk model outperformed conventional ICU severity scores in identifying high-risk sepsis-associated pneumonia patients. Our novel nomogram demonstrated superior predictive performance for 28-day mortality, achieving area under the curve (AUC) values of 0.71 (training), 0.70 (internal validation), and 0.70 (external validation), consistently exceeding the performance of conventional ICU severity scores.

**Conclusion:**

Our multicenter study demonstrates a consistent inverse association between GNRI and short-term mortality in critically ill patients with sepsis-associated pneumonia across both cohorts. These findings position GNRI as a practical, readily available risk-stratification tool that may assist clinicians in promptly identifying high-risk patients for targeted nutritional interventions and intensified monitoring.

## Introduction

Sepsis-associated pneumonia represents a formidable challenge in intensive care units, contributing significantly to global morbidity and mortality despite advances in antimicrobial therapy and supportive care (1, 2). The convergence of population aging, antimicrobial resistance, and the persistent threat of emerging pathogens has sustained ICU mortality rates for this condition at alarmingly high levels, particularly among patients requiring invasive mechanical ventilation (3, 4). Accurate prognostication remains crucial for risk stratification and resource allocation, yet conventional approaches display notable limitations. Widely-used scoring systems (e.g., PSI, CURB-65), often derived from retrospective cohorts, may systematically underestimate illness severity in younger patients or special populations (e.g., the immunocompromised), while routine biomarkers such as PCT and CRP lack specificity and are frequently confounded by non-infectious inflammatory states (5–8). This persistent prognostic uncertainty underscores the critical need for novel, reliable, and readily available tools to guide clinical decision-making.

In recent years, the pivotal role of nutritional status in modulating immune responses and determining clinical outcomes in critical illness has gained increasing recognition (9–11). Malnutrition, prevalent among critically ill patients, initiates a cascade of immunosuppressive effects. Protein-energy deficits impair lymphocyte proliferation and function, diminish complement and immunoglobulin production, and compromise the integrity of mucosal and epithelial barriers, facilitating bacterial translocation (12, 13). Concurrently, deficiencies in key micronutrients (e.g., zinc, selenium, vitamins) further disrupt oxidative stress responses and immune cell signaling (14). This state of immune dysfunction exacerbates the vulnerability to infectious complications and hampers the resolution of inflammation, thereby creating a vicious cycle that profoundly impacts recovery and survival (15). The Geriatric Nutritional Risk Index (GNRI), a simple objective tool calculated from serum albumin and body weight, has consequently emerged as a robust prognostic biomarker. Its utility extends beyond geriatrics, demonstrating significant predictive value for mortality and complications across a spectrum of conditions, including cardiovascular diseases, malignancies, and other critical illnesses (16, 17). Importantly, this predictive utility has been confirmed in heterogeneous, broad critical care populations, underscoring its role as a general marker of physiological reserve in the acutely ill (18). The relevance of the GNRI in sepsis is underpinned by the specific pathophysiology of this condition. Sepsis triggers a severe hypercatabolic state, driving a profound negative protein-energy balance. Serum albumin levels decline due to a combination of reduced hepatic synthesis, increased vascular permeability, and hemodilution, serving as a marker of systemic inflammation and visceral protein depletion. Concurrently, a low body weight or body mass index reflects diminished somatic protein and energy reserves (19, 20). Thus, the GNRI directly quantifies the central nutritional derangement in sepsis—protein-energy wasting—which exacerbates immune dysfunction and organ failure. This suggests that GNRI captures a fundamental aspect of physiological reserve and resilience that is critical in sepsis.

However, a targeted investigation into the prognostic value of GNRI specifically in the high-risk population of critically ill patients with sepsis-associated pneumonia is lacking. The pathophysiological interplay between nutritional status, immune competence, and sepsis progression is particularly salient in this context, yet it remains an underexplored nexus in prognostic research (21, 22). To address this knowledge gap, we conducted a retrospective cohort study leveraging the large-scale Medical Information Mart for Intensive Care IV (MIMIC-IV) database as a derivation cohort, complemented by an external validation cohort. To comprehensively evaluate the relationship between GNRI and short-term prognosis of sepsis associated pneumonia, and to provide a clinically feasible strategy for improving risk stratification and personalized management of this vulnerable patient population.

## Materials and methods

### Data source

We conducted a retrospective study using two distinct patient cohorts to ensure the robustness of our findings. Our primary analysis was performed on data from the MIMIC-IV database (23, 24), which contains comprehensive, de-identified clinical information from more than 190,000 adult ICU patients at a large U.S. academic medical center (2008–2019). This cohort served as our derivation cohort. To confirm that our results are generalizable beyond a single database, we established an external validation cohort. This group consisted of consecutive patients with sepsis-associated pneumonia treated in the ICUs of Jinyang Hospital, Guizhou Medical University (China) from March 2023 to March 2025. The study protocol was approved by the Ethics Committee of Jinyang Hospital, Guizhou Medical University (JYYY-2025-WZ-23). The requirement for individual patient informed consent was waived due to the retrospective nature of the study and the use of anonymized data.

### Study population

This study specifically examined patients with “sepsis-associated pneumonia” during their initial ICU admission. “sepsis-associated pneumonia” was defined as the co-occurrence of pneumonia and sepsis in a critically ill patient, with pneumonia considered the likely source of infection. Meanwhile sepsis was defined according to the Sepsis-3 criteria as a life-threatening organ dysfunction caused by a dysregulated host response to infection, operationalized as an acute increase in the Sequential Organ Failure Assessment (SOFA) score by ≥ 2 points. Pneumonia diagnoses were classified using ICD-9/10 codes, including pneumococcal pneumonia, Klebsiella pneumonia, fungal pneumonia, viral pneumonia, gram-negative bacterial pneumonia, aspiration pneumonia, unspecified pneumonia, and related pulmonary infections. Inclusion criteria comprised: (1) age ≥65 years; (2) survival and ICU stay duration ≥24 h; (3) complete anthropometric measurements (weight, height) and serum albumin records; (4) first-time ICU admission. (5) All clinical data were collected within the initial 24 h of ICU admission. (6) SOFA score ≥ 2 points.

### Variable extraction

Data extraction was performed using PostgreSQL (v13.7.2) and Navicat Premium (v16) with structured query language (SQL). Extracted variables were categorized into six domains: (1) Demographic characteristics: age, sex, weight, height, race, and BMI; (2) Comorbidities: hypertension, AKI, CKD, HF, diabetes mellitus, HLD, CB, and COPD; (3) Vital signs: respiratory rate (RR), heart rate (HR), non-invasive blood pressure (mean [NBPM], systolic [NBPS], diastolic [NBPD]), and oxygen saturation (SpO_2_); (4) Severity scores: APS IIII, SAPS III, OASIS, SOFA and APACHE II; (5) Laboratory parameters: lymphocyte count, RBC, WBC, neutrophils, hemoglobin, platelet count, RDW, HCT, albumin, anion gap, lactate, sodium, calcium, potassium, chloride, TCO_2_, pO_2_, uric acid, LDH, pH, creatinine, BUN, INR, PT, aPTT, ALT, AST, and total bilirubin; (6) Interventions: mechanical ventilation and CRRT; To minimize bias, variables with >30% missing data were excluded, while those with ≤ 30% missingness underwent multiple imputation.

### Definition of GNRI and endpoint

The Geriatric Nutritional Risk Index (GNRI) was employed as the primary nutritional assessment tool. The GNRI is a simple, objective index composed of two routinely measured clinical parameters: serum albumin concentration and body weight. It was calculated for each patient using the established formula: GNRI = [1.489 × serum albumin (g/L)] + (41.7 × [current body weight/ideal body weight]) (15). For this calculation, the serum albumin value (in g/L) was obtained from the first laboratory measurement within 24 h of ICU admission. The ideal body weight was derived from a standard ideal Body Mass Index (BMI) of 22 kg/m^2^, calculated as ideal body weight (kg) = 22 × (height in meters)^2^. Based on the calculated GNRI score, patients were stratified into four predefined nutritional risk categories that have been validated for prognostic assessment: no nutritional risk (GNRI ≥ 98), low nutritional risk (92 ≤ GNRI < 98), moderate nutritional risk (82 ≤ GNRI < 92), and high nutritional risk (GNRI < 82). This stratification directly links the index score to clinically meaningful risk levels, allowing us to investigate the graded association between nutritional status, as quantified by the GNRI, and patient mortality. The primary endpoint was 28-day ICU mortality, with 28-day in-hospital all-cause mortality serving as the secondary endpoint.

### Risk prediction modeling and validation

In the internal cohort, patients were randomly allocated to test and train cohort in a 7:3 ratio. This commonly used ratio provides a sufficient sample size in the test set for stable model development and parameter estimation, while reserving an adequate, independent subset for unbiased internal validation of model performance. This approach helps to mitigate overfitting and assess the generalizability of the model within the same population (25). We then performed variable selection using the Least Absolute Shrinkage and Selection Operator (LASSO) regression, implemented with the R package “glmnet.” The purpose of LASSO regression is to identify the most parsimonious set of predictors by penalizing model complexity, which helps to prevent overfitting—a scenario where a model performs well on the current data but poorly on new data. This approach is particularly valuable when dealing with a large number of potential predictor variables, as it automatically shrinks the coefficients of less important variables to zero, effectively selecting only the key features most strongly associated with the outcome. The optimal penalty parameter (λ) was selected via 10-fold cross-validation to ensure robustness (26, 27). Subsequently, the variables selected by LASSO were entered into a multivariable Cox proportional hazards regression model to confirm their statistical significance and to estimate their effect sizes for model construction. This final model was visualized as a nomogram. Model performance was ultimately evaluated across all three cohorts (test cohort, train cohort, and external cohort) using time-dependent receiver operating characteristic (ROC) analysis, with predictive accuracy quantified by the area under the curve (AUC).

### Statistical analysis

Continuous variables are presented as medians with interquartile ranges (IQR) or mean ± standard deviation (SD). Group comparisons were performed using Student's *t*-test or ANOVA for normally distributed data and Mann-Whitney *U* or Kruskal-Wallis tests for non-normally distributed data. Categorical variables are expressed as counts (percentages) and compared using Pearson's χ^2^ test or Fisher's exact test, as appropriate. Univariate regression identified variables associated with 28-day mortality. Multivariable Cox proportional hazards models then assessed the independent prognostic value of GNRI, reporting hazard ratios (HRs) with 95% confidence intervals (Cis). Three adjustment models were constructed: Model 1 (unadjusted); Model 2 (adjusted for demographics and comorbidities); Model 3 additionally adjusted for vital signs, interventions, and significant laboratory parameters (Supplementary Tables S1–S3). We evaluated trend associations and calculated variance inflation factors (VIF) to assess multicollinearity. Restricted cubic splines with four knots (at the 5th, 35th, 65th, and 95th percentiles) examined GNRI-outcome relationships after full covariate adjustment. All analyses were conducted using R software (version 4.2.2). A two-sided *p*-value < 0.05 defined statistical significance.

## Result

### Baseline characteristics of study participants

**Figure 1:**
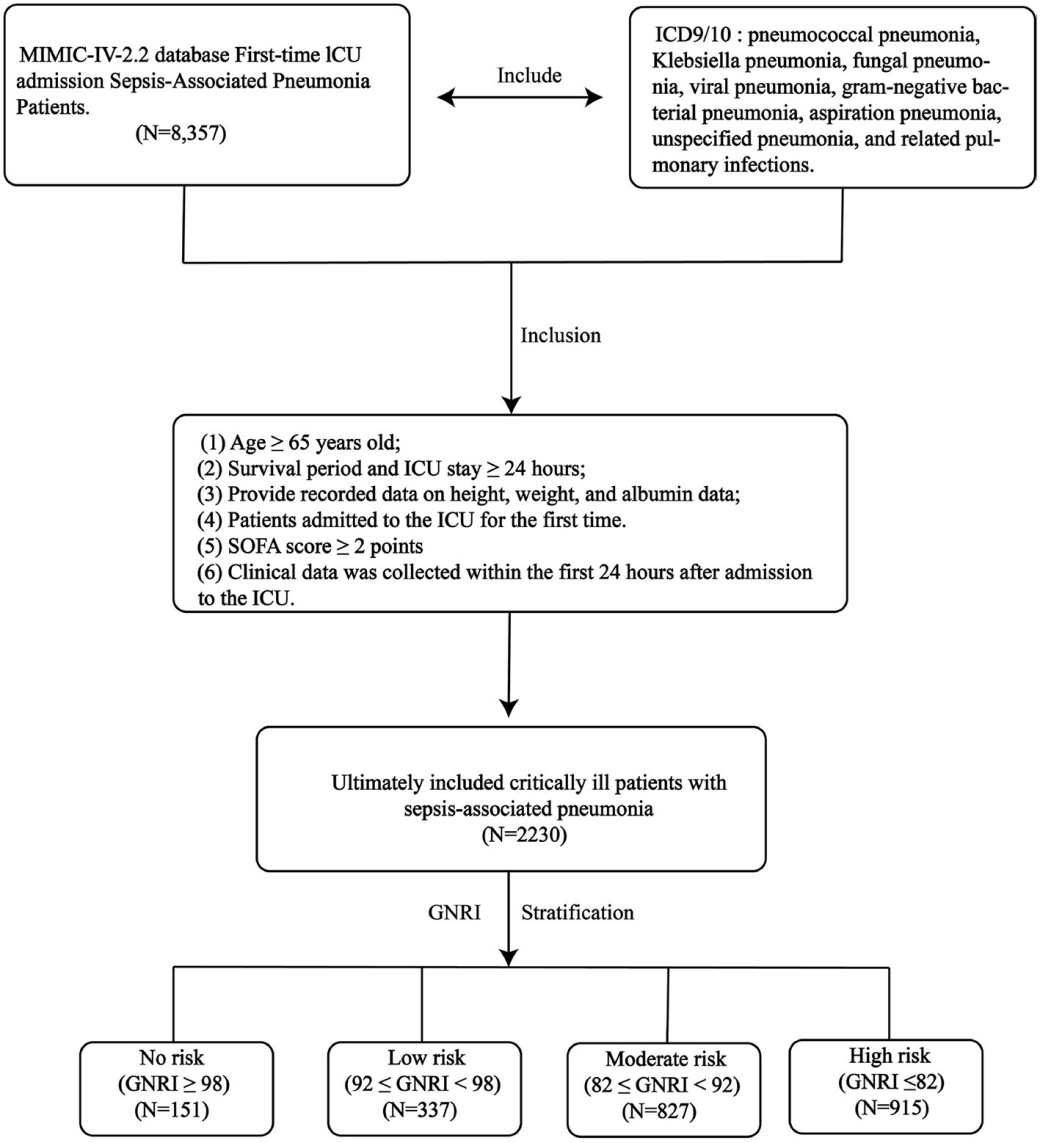
The research design and workflow of this study.

After applying inclusion/exclusion criteria, we analyzed 2,230 critically ill patients with sepsis-associated pneumonia from the MIMIC-IV database (Figure 1). The 28-day ICU and in-hospital mortality rates were 26.64% and 26.59%, respectively. Table 1 details the baseline characteristics stratified by GNRI categories. The cohort had a median age of 77 years (IQR: 65–102) with 1,317 (59.06%) male patients. Common comorbidities included acute kidney injury (69.89%), heart failure (41.70%), chronic bronchitis (20.09%), and COPD (23.36%). GNRI stratification identified high-risk (*n* = 915, 41.03%), moderate-risk (*n* = 827, 37.09%), low-risk (*n* = 337, 15.11%), and no-risk (*n* = 151, 6.77%) groups. Patients with nutritional risk (lower GNRI) were typically older and had lower BMI. Lower GNRI values correlated with higher severity scores (SOFA, APS IIII, SAPS III, OASIS, and APACHE II). Compared to the no-risk group, high-risk patients showed elevated heart rate, respiratory rate, RDW, WBC, neutrophils, lactate, and pH, but lower hemoglobin, pO_2_, RBC, albumin, haematocrit, and blood pressure. The high-risk group had significantly greater mortality than the no-risk group (28-day ICU mortality: 34.1 vs. 12.58%, *p* < 0.001; overall 28-day mortality: 34.21 vs. 11.92%, *p* < 0.001).

**Table 1:** Summary descriptive table by groups of GNRI score group.

**Variable**	**ALL**	**High**	**Moderate**	**Low**	**No**	***p* Overall**
	***N** =* **2,230**	***N** =* **915**	***N** =* **827**	***N** =* **337**	***N** =* **151**	
GNRI	84.88 (50.63–116.15)	76.39 (50.63–81.98)	86.37 (82.05–91.74)	93.82 (92.12–97.98)	100.9 (98.28–116.15)	< 0.001
**Gender**						
F	913 (40.9%)	405 (44.3%)	321 (38.8%)	138 (40.9%)	49 (32.5%)	0.017
M	1,317 (59.1%)	510 (55.7%)	506 (61.2%)	199 (59.1%)	102 (67.5%)	
Age	77 (65–102)	69 (65–92)	80 (74–85)	88 (85–91)	92 (91–102)	< 0.001
BMI	27.27 (11.73–108.69)	26.06 (11.73–93.3)	27.79 (15.09–108.69)	28.41 (15.37–92.54)	28.77 (19.65–59.28)	< 0.001
**Race**						
White	1,221 (54.75%)	516 (56.39%)	445 (53.81%)	191 (56.68%)	69 (45.70%)	0.079
Other race	1,009 (45.25%)	399 (43.61%)	382 (46.19%)	146 (43.32%)	82 (54.30%)	
**HYPT**						
Yes	636 (28.5%)	264 (28.9%)	222 (26.8%)	95 (28.2%)	55 (36.4%)	0.120
No	1,594 (71.48%)	651 (71.15%)	605 (73.16%)	242 (71.81%)	96 (63.58%)	
**AKI**						
Yes	1,447 (64.89%)	614 (67.10%)	529 (63.97%)	221 (65.58%)	83 (54.97%)	0.031
No	783 (35.11%)	301 (32.90%)	298 (36.03%)	116 (34.42%)	68 (45.03%)	
**LIC**						
Yes	274 (12.29%)	125 (13.66%)	79 (9.55%)	47 (13.95%)	23 (15.23%)	0.024
No	1,956 (87.71%)	790 (86.34%)	748 (90.45%)	290 (86.05%)	128 (84.77%)	
**CKD**						
Yes	652 (29.24%)	242 (26.45%)	269 (32.53%)	106 (31.45%)	35 (23.18%)	0.010
No	1,578 (70.76%)	673 (73.55%)	558 (67.47%)	231 (68.55%)	116 (76.82%)	
**Diabetes**						
Yes	755 (33.86%)	313 (34.21%)	286 (34.58%)	112 (33.23%)	44 (34.48%)	0.733
No	1,575 (66.14%)	602 (65.79%)	541 (65.42%)	178 (66.77%)	84 (65.62%)	
**Hld**						
Yes	855 (38.34%)	300 (32.79%)	343 (41.48%)	156 (46.29%)	56 (37.09%)	< 0.001
No	1,375 (61.66%)	615 (67.21%)	484 (58.52%)	181 (53.71%)	95 (62.91%)	
**Cb**						
Yes	448 (20.09%)	184 (20.11%)	152 (18.38%)	87 (25.82%)	25 (16.56%)	0.023
No	1,782 (79.91%)	731 (79.89%)	675 (81.62%)	250 (74.18%)	126 (83.44%)	
**HF**						
Yes	930 (41.70%)	315 (34.43%)	391 (47.28%)	166 (49.26%)	58 (38.41%)	< 0.001
No	1,300 (58.30%)	600 (65.57%)	436 (52.72%)	171 (50.74%)	93 (61.59%)	
**IHD**						
Yes	906 (40.63%)	345 (37.70%)	356 (43.05%)	142 (42.14%)	63 (41.72%)	0.130
No	1,324 (59.37%)	570 (62.30%)	471 (56.95%)	195 (57.86%)	88 (58.28%)	
**COPD**						
Yes	521 (23.36%)	212 (23.17%)	182 (22.01%)	99 (29.38%)	28 (18.54%)	0.022
No	1,709 (76.64%)	703 (76.83%)	645 (77.99%)	238 (70.62%)	123 (81.46%)	
**MV**						
Yes	2,053 (92.06%)	825 (90.16%)	768 (92.87%)	322 (95.55%)	138 (91.39%)	0.012
No	177 (7.94%)	90 (9.84%)	59 (7.13%)	15 (4.45%)	13 (8.61%)	
**CRRT**						
Yes	288 (12.91%)	146 (15.96%)	98 (11.85%)	33 (9.79%)	11 (7.28%)	0.001
No	1,942 (87.09%)	769 (84.04%)	729 (88.15%)	304 (90.21%)	140 (92.72%)	
SOFA	7 (2–21)	8 (2–21)	7 (2–20)	6 (2–18)	6 (2–21)	< 0.001
APSII	53 (9–184)	59 (12–184)	51 (10–162)	47 (9–125)	47 (13–139)	< 0.001
SAPSII	43 (6–112)	46 (6–112)	43 (6–100)	39 (12–96)	36 (10–81)	< 0.001
OASIS	36 (12–64)	38 (16–62)	36 (12–64)	33 (17–54)	34 (14–54)	< 0.001
APACHEII	21 (1–53)	22 (4–52)	21 (3–53)	20 (1–42)	19 (2–45)	< 0.001
Lym	0.94 (0–165.72)	0.86 (0–46.28)	0.97 (0–165.72)	0.96 (0–10.84)	1.18 (0.12–5.24)	< 0.001
HCT	31.8 (13–64.6)	29.7 (13–64.6)	32.6 (14.3–54.3)	34.3 (15.9–60.4)	37.5 (19.4–53)	< 0.001
Hb	10.1 (4.1–19.8)	9.4 (4.1–19.8)	10.3 (4.8–17.8)	10.9 (5.3–19.1)	12.2 (6.1–17.2)	< 0.001
PLT	185 (9–1,647)	183 (9–1,379)	181 (12–991)	196 (16–568)	196 (18–1,647)	0.740
RDW	15.4 (11.4–33.1)	15.9 (11.8–28.1)	15.2 (11.6–29.2)	14.8 (11.6–33.1)	14.5 (11.4–26.5)	< 0.001
Red	3.41 (1.46–6.82)	3.21 (1.46–6.58)	3.53 (1.55–6.82)	3.72 (1.47–6.24)	4.09 (1.84–6.34)	< 0.001
WBC	12.3 (0.1–321.1)	12.7 (0.2–87.1)	12.2 (0.1–321.1)	11.6 (0.5–88.8)	12.2 (1.3–58.2)	0.332
Neu	9.945 (0–79.7)	10.47 (0–79.7)	9.79 (0.06–60.44)	9.5 (0.26–75.55)	9.49 (0.77–57.22)	0.028
ALB	2.9 (0.6–5)	2.4 (0.6–3.5)	3 (2.8–4)	3.5 (3.4–4.3)	4 (3.8–5)	< 0.001
AG	15 (3–47)	15 (3–47)	15 (5–41)	15 (6–33)	16 (7–42)	0.005
Ca	8.3 (4.2–14.1)	8 (4.3–14.1)	8.4 (4.2–12.5)	8.7 (5.6–11.1)	8.8 (7.1–12.2)	< 0.001
Cl	102 (61–139)	103 (61–135)	102 (69–139)	102 (73–123)	101 (64–117)	< 0.001
Potassium	4.2 (2.3–8.9)	4.2 (2.3–7.8)	4.2 (2.6–8.9)	4.3 (2.4–7.4)	4.1 (2.4–7.5)	0.235
Sodium	138 (100–175)	138 (100–165)	138 (111–175)	139 (116–157)	138 (111–158)	0.240
TCO2	24 (0–59)	24 (6–59)	25 (8–55)	25 (0–48)	24 (7–59)	< 0.001
Lac	1.8 (0.3–22)	2.1 (0.5–22)	1.7 (0.3–17.4)	1.7 (0.5–10.6)	2 (0.6–17)	< 0.001
pco2	43 (18–106)	43 (20–102)	43 (20–105)	43 (18–106)	41 (20–89)	0.057
ph	7.35 (6.84–7.65)	7.35 (6.84–7.65)	7.36 (6.88–7.58)	7.36 (6.95–7.58)	7.37 (6.93–7.51)	0.002
po2	63 (16–681)	61 (18–681)	67 (16–459)	62 (20–514)	73 (21–504)	0.051
INR	1.3 (0.9–13.8)	1.4 (0.9–13.8)	1.3 (0.9–11.1)	1.3 (1–10.8)	1.3 (0.9–10.9)	< 0.001
PT	14.7 (9.4–150)	15.4 (9.5–150)	14.3 (9.4–150)	13.8 (10.4–118)	14.1 (9.8–119.2)	< 0.001
APTT	31.5 (17.8–150)	32.3 (19.2–150)	30.8 (19.8–150)	31 (17.8–150)	32.4 (21.4–150)	0.005
TG	148 (5–6,219)	150 (11–3,668)	149 (14–6,219)	146 (5–1,487)	140 (20–757)	0.369
ALT	26 (4–15,018)	25 (4–6,026)	26 (4–7,805)	25 (5–4,687)	27 (5–15,018)	0.173
AST	40 (6–15,108)	41 (6–11,380)	37 (6–14,244)	39 (8–7,638)	43 (10–15,108)	0.224
TB	0.6 (0.1–51.2)	0.7 (0.1–33.7)	0.6 (0.1–40.7)	0.6 (0.2–38.5)	0.7 (0.2–51.2)	0.001
CRE	1.3 (0.2–32)	1.3 (0.2–13.5)	1.3 (0.3–19.1)	1.3 (0.3–32)	1.2 (0.5–9)	0.445
UREA	27 (2–227)	29 (2–213)	27 (3–227)	25 (7–194)	20 (4–155)	0.003
UC	6.3 (1.3–26.6)	6.1 (1.3–26.6)	6.3 (1.3–18.5)	6.5 (1.3–13.1)	6.5 (1.4–13.1)	0.212
LDH	325 (78–19,980)	335 (87–19,980)	323 (78–10,955)	312 (123–9,290)	297 (93–10,530)	0.572
HR	92 (0–182)	94 (0–182)	91 (37–166)	89 (28–162)	87 (0–164)	< 0.001
NBPS	118 (35–216)	112 (35–216)	120 (51–207)	123 (57–203)	127 (45–194)	< 0.001
NBPD	68 (11–158)	66 (11–154)	69 (18–158)	70 (32–144)	72 (30–129)	< 0.001
NBPM	82 (15–167)	79 (15–167)	83 (19–166)	84 (45–155)	86 (37–137)	< 0.001
RR	21 (0–76)	22 (0–58)	20 (0–73)	20 (5–76)	20 (7–46)	0.006
Spo2	97 (39–100)	97 (58–100)	97 (61–100)	97 (39–100)	97 (84–100)	0.555
Hosp_28_time	12.77 (0.24–27.95)	13.26 (0.24–27.95)	12.44 (0.94–27.94)	12.06 (0.51–27.87)	12.71 (1.68–27.92)	0.261
ICU_28_time	4.52 (0.02–27.91)	4.13 (0.02–27.91)	4.75 (0.03–26.72)	4.56 (0.22–27.08)	4.96 (0.3–24.81)	0.568
Hosp_28	593 (26.59%)	313 (34.21%)	204 (24.67%)	58 (17.21%)	18 (11.92%)	< 0.001
ICU_28	594 (26.64%)	312 (34.10%)	205 (24.79%)	58 (17.21%)	19 (12.58%)	< 0.001

HYPT, hypertension; AKI, acute kidney injury; LIC, liver cirrhosis; CKD, chronic kidney disease; Hld, hyperlipidemia; CB, chronic bronchitis; HF, heart failure; COPD, chronic obstructive pulmonary disease; BMI, body mass index; SOFA, sequential organ failure assessment; APSIII, acute physiology and chronic health III score; SAPSII, simplified acute physiology score II; OASIS, oxford acute severity of illness score; APACHII, acute physiology and chronic health evaluation II; HR, heart rate; NBPS, non-invasive blood pressure systolic; RR, respiratory rate; NBPM, non-invasive blood pressure mean; NBPD, non invasive diastolic blood pressure; Lym, lymphocyte; HCT, hematocrit; Hb, hemoglobin; PLT, platelet; RDW, red blood cell distribution width; WBC, white blood cell; Neu, neutrophil; Mon, monocyte; ALB, albumin; AG, anion gap; K, potassium; Na, sodium; LAC, lactate; INR, international normalized ratio; PT, prothrombin time; APTT, activated partial thromboplastin time; ALT, alanine aminotransferase; AST, aspartate aminotransferase; TB, total bilirubin; CRE, creatinine; UREA, urea nitrogen; CRRT, continuous renal replacement therapy; MV, mechanical ventilation.

### Multivariate Cox regression analysis of the correlation between GNRI and short-term mortality in critically ill sepsis pneumonia patients

**Figure 2:**
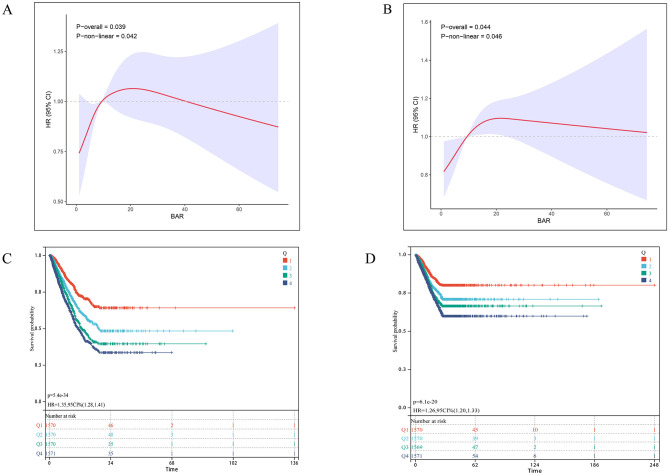
Association between GNRI and 28-day survival in the internal cohort. **(A, B)** Restricted cubic spline (RCS) curves show the nonlinear relationship between continuous GNRI score and the odds of **(A)** 28-day ICU mortality and **(B)** 28-day in-hospital mortality. The solid line represents the adjusted odds ratio (OR), with the dashed lines indicating the 95% confidence interval. **(C, D)** Kaplan-Meier survival curves illustrate the probability of survival from **(C)** ICU death and **(D)** in-hospital death 28 days, stratified by GRNI score stratification (No nutritional risk, blue; Low nutritional risk, green; Moderate nutritional risk, dark green; High nutritional risk, red). *p*-Value and Hazard ratios (HR) with 95% confidence intervals are derived from Cox proportional hazards models, using the Low GNRI group as reference.

As shown in Table 2, the fully adjusted multivariable Cox proportional hazards model demonstrated a significant inverse association between higher GNRI scores and 28-day ICU mortality (HR = 0.99, 95% CI: 0.98–1.00). Compared to the high nutritional risk group, both low (HR = 0.69, 95% CI: 0.51–0.93) and no (HR = 0.41, 95% CI: 0.25–0.69) nutritional risk groups showed significantly lower mortality risks. Similarly, for 28-day in-hospital all-cause mortality, the no nutritional risk group had significantly lower mortality than the high-risk group (HR = 0.59, 95% CI: 0.36–0.98). Trend tests confirmed a dose-response relationship between increasing nutritional risk and both ICU/hospital mortality (*p* < 0.05 for trend). Restricted cubic spline analysis revealed an inverse dose-response relationship between GNRI and mortality (nonlinear *p* = 0.013 for ICU mortality; *p* = 0.034 for hospital mortality; Figures 2A, B). Kaplan-Meier survival curves further validated the significant inverse correlation between higher GNRI scores and reduced mortality. Compared to low GNRI groups, high GNRI was associated with lower 28-day ICU mortality (HR = 0.71, 95% CI: 0.64–0.79) and hospital mortality (HR = 0.74, 95% CI: 0.67–0.82; Figures 2C, D).

**Table 2:** The relationship between GNRI score and 28 days ICU/hospital mortality rate.

**Characteristic**	**Model 1**			**Model 2**			**Model 3**		
	**HR**	**95% CI**	***p*** **-Value**	**HR**	**95% CI**	***p*** **-Value**	**HR**	**95% CI**	***p*** **-Value**
GNRI	0.97	0.96, 0.98	< 0.001	0.97	0.97, 0.98	< 0.001	0.99	0.98, 1.00	0.008
**GNRI group**									
High risk	Ref	Ref		Ref	Ref		Ref	Ref	
Moderate risk	0.73	0.61, 0.87	< 0.001	0.79	0.66, 0.94	0.009	0.89	0.74, 1.09	0.3
Low risk	0.53	0.40, 0.70	< 0.001	0.53	0.40, 0.71	< 0.001	0.69	0.51, 0.93	0.017
No risk	0.33	0.21, 0.52	< 0.001	0.39	0.25, 0.63	< 0.001	0.41	0.25, 0.69	< 0.001
GNRI	0.97	0.97, 0.98	< 0.001	0.98	0.97, 0.99	< 0.001	0.99	0.98, 1.00	0.2
**GNRI group**									
High risk	Ref	Ref		Ref	Ref		Ref	Ref	
Moderate risk	0.75	0.63, 0.90	0.002	0.82	0.68, 0.98	0.027	0.97	0.80, 1.17	0.8
Low risk	0.58	0.44, 0.76	< 0.001	0.63	0.47, 0.83	0.001	0.83	0.61, 1.12	0.2
No risk	0.37	0.23, 0.60	< 0.001	0.46	0.28, 0.74	0.001	0.59	0.36, 0.98	0.042

HR, hazard ratio; CI, confidence interval.

### Incremental prognostic value of GNRI score

In the internal cohort, we evaluated the incremental predictive value of adding GNRI to conventional severity scores (APACHE II, SOFA, APS IIII, SAPS III and OASIS) for 28-day ICU and in-hospital mortality using area under the curve (AUC) analysis. As shown in Figures S1, S2, incorporating GNRI improved predictive accuracy across all models for 28-day ICU mortality: APACHE II (AUC: 0.63–0.64), SOFA (0.63–0.67), APS IIII (0.66–0.68), SAPS III (0.66–0.68) and OASIS (0.63–0.66). Similar improvements were observed for 28-day in-hospital mortality: APACHE II (0.63–0.67), SOFA (0.63–0.66), APS IIII (0.66–0.69), SAPS III (0.66–0.68) and OASIS (0.63–0.66).

### External cohort verification

**Figure 3:**
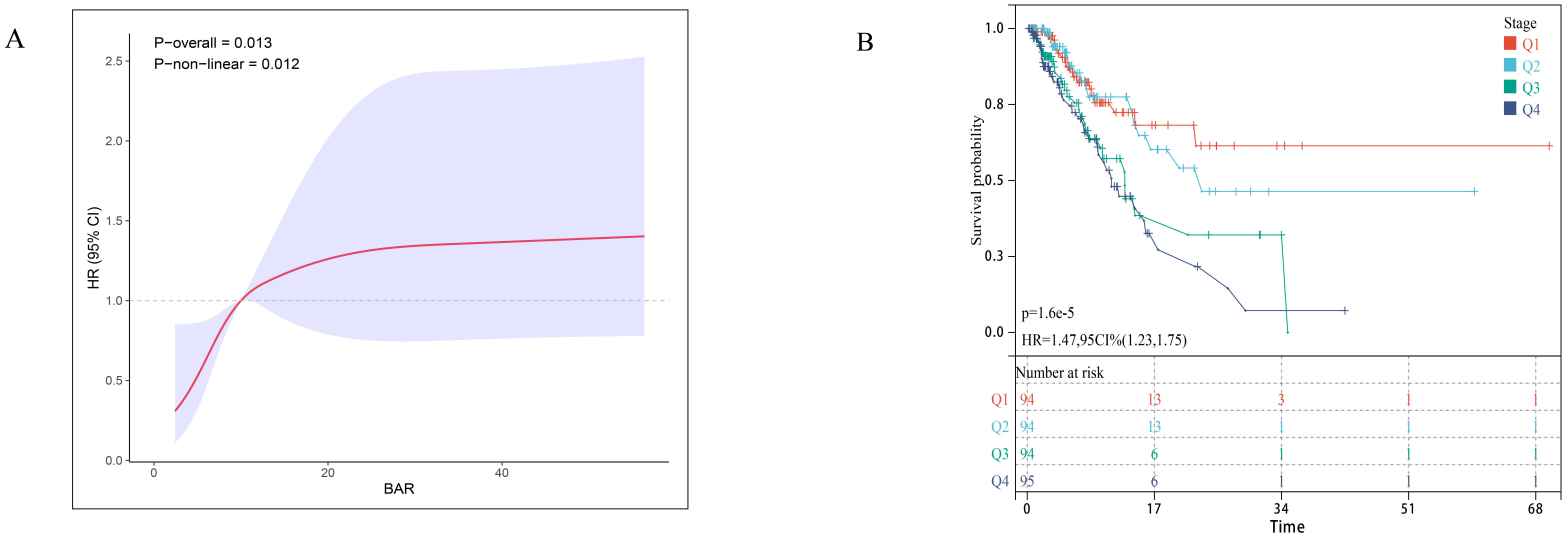
Association between GNRI and 28-day survival in the external validation cohort. **(A)** Restricted cubic spline plot showing the dose-response relationship between continuous Geriatric Nutritional Risk Index (GNRI) values and 28-day in-hospital mortality in the external validation cohort. **(B)** Kaplan-Meier survival curves illustrate the probability of survival from ICU death 28 days, stratified by GRNI score stratification.

To further validate the GNRI-mortality association, we tested the model in an external cohort of 245 ICU patients with sepsis-associated pneumonia, which demonstrated a 28-day ICU mortality rate of 28.57% (Supplementary Table S3). The adjusted multivariable Cox model confirmed a significant inverse association between higher GNRI scores and mortality (HR = 0.97, 95% CI: 0.95–1.00). Compared to the high nutritional risk group, no nutritional risk (low GNRI) showed a particularly strong mortality association (HR = 0.17, 95% CI: 0.05–0.58; Table 3). Restricted cubic splines revealed an inverse dose-response relationship (nonlinear *p* = 0.013) in the external cohort (Figure 3A). Kaplan-Meier analysis demonstrated significantly reduced mortality with higher GNRI (HR = 0.62, 95% CI: 0.48–0.81; Figure 3B).

**Table 3:** The relationship between GNRI score and external verification cohort 28 days ICU mortality rate.

**Characteristic**	**Model 1**			**Model 2**			**Model 3**		
	**HR**	**95% CI**	***p*** **-Value**	**HR**	**95% CI**	***p*** **-Value**	**HR**	**95% CI**	***p*** **-Value**
GNRI	0.96	0.94, 0.98	< 0.001	0.97	0.95, 0.99	0.004	0.97	0.95, 1.00	0.035
**GNRI group**									
High risk	—	—		—	—		—	—	
Moderate risk	0.56	0.33, 0.96	0.034	0.63	0.36, 1.09	0.10	0.69	0.37, 1.30	0.3
Low risk	0.47	0.24, 0.92	0.028	0.49	0.25, 0.96	0.038	0.59	0.27, 1.29	0.2
No risk	0.19	0.07, 0.55	0.002	0.26	0.09, 0.78	0.016	0.17	0.05, 0.58	0.005

HR, hazard ratio; CI, confidence interval.

### Line chart and risk model construction

**Figure 4:**
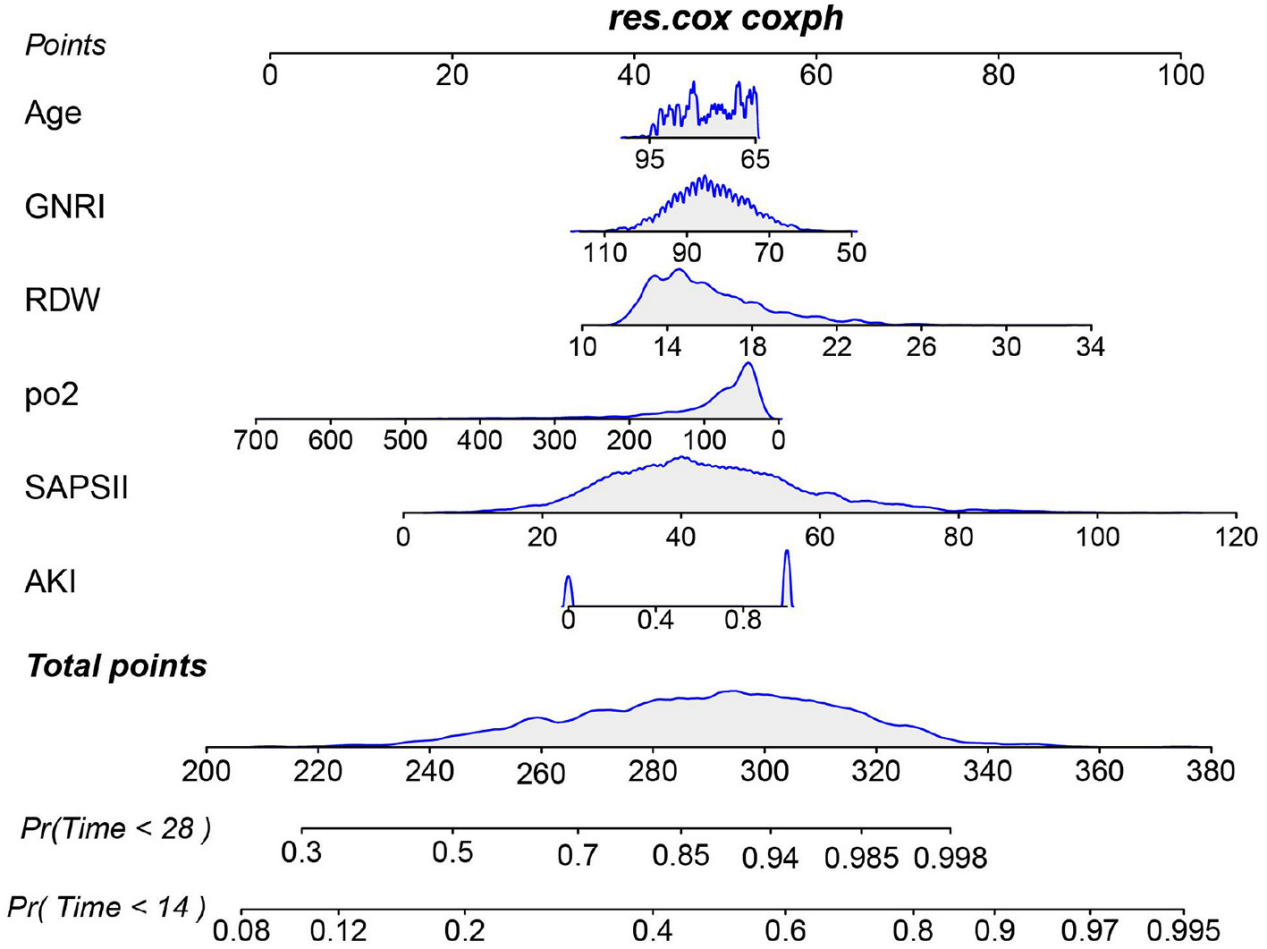
Nomogram for predicting the risk of 28-day ICU mortality. This clinical tool integrates the GNRI score and other independent predictors identified from multivariate analysis. To use the nomogram: for each patient variable, locate the value on the corresponding axis and draw a line upward to the “Points” axis to determine the individual score. Sum the points for all variables, locate the total on the “Total Points” axis, and then draw a line straight down to the “Risk of 28-day ICU mortality” axis to obtain the individualized predicted probability.

**Figure 5:**
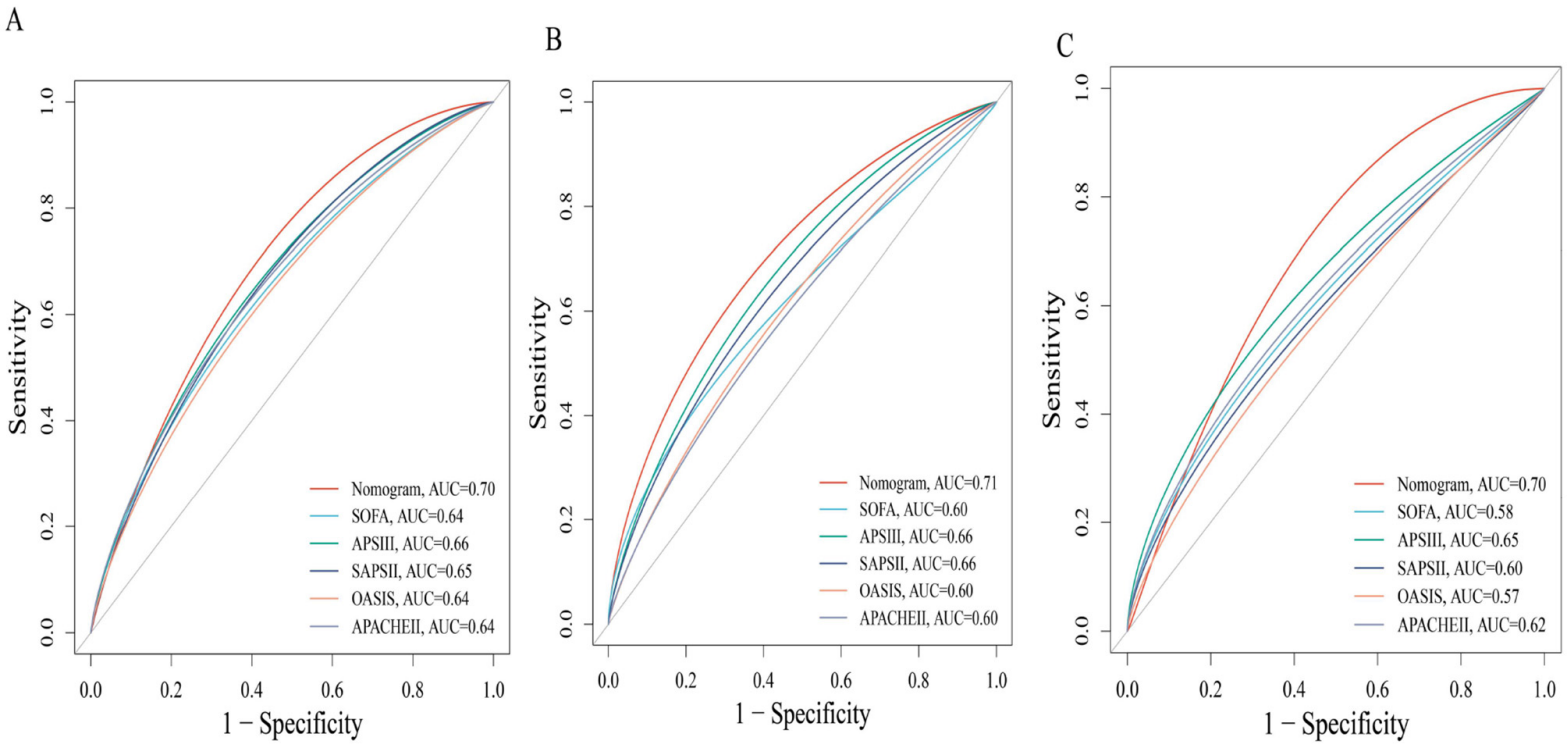
Receiver operating characteristic (ROC) curves comparing the predictive performance of the novel nomogram and conventional severity scores for 28-day mortality. **(A)** Test cohort 1; **(B)** train cohort 2; **(C)** external verification cohort.

We first identified variables significantly associated with 28-day ICU mortality through univariate Cox regression across all three cohorts (training, testing, and external validation). Venn diagram analysis revealed 13 shared prognostic variables, which were subsequently refined to six core predictors via LASSO regression (Figure S3). Multivariable Cox regression ultimately identified six independent predictors—GNRI, age, RDW, pO_2_, SAPS III, and AKI – for constructing our 28-day ICU mortality risk model (Figure 4 and Figure S4). Compared to conventional severity scores (SOFA, APS IIII, SAPS III, OASIS), our nomogram demonstrated superior sensitivity and specificity, with AUROCs of 0.70 (testing cohort; Figure 5A), 0.71 (training cohort; Figure 5B) and 0.70 (external cohort; Figure 5C).

## Discussion

In this retrospective two-center study, we analyzed 2,230 critically ill patients with sepsis-associated pneumonia from an internal derivation cohort to investigate the association between the Geriatric Nutritional Risk Index (GNRI) and short-term (28-day) mortality, with findings further validated in an independent external cohort of 245 patients. This association remained robust after extensive adjustment for severity of illness and comorbidities, suggesting that GNRI captures a dimension of physiological frailty that is not fully accounted for by traditional critical care scoring systems.

Different from the Prognostic Nutritional Index (PNI), Controlled Nutritional Status (CONUT) score, hemoglobin, albumin, lymphocyte, and platelet (HALP) score and Naples Prognostic Score (NPS). The novelty and clinical utility of our work lie in the specific validation of the GNRI in sepsis-pneumonia. Our comparative ROC analysis (Figure S5) indicates that GNRI offered prognostic discrimination comparable to or better than PNI and HALP in our cohorts. In contrast to the more immune-centric PNI or the complex CONUT and NPS, the GNRI's simplicity—relying solely on albumin and body weight—is its greatest asset in the time-sensitive ICU environment (28–33). This parsimony, however, does not diminish its power. By focusing on albumin (a marker of visceral protein stores and inflammatory burden) and body weight (a surrogate for somatic protein reserves), the GNRI directly targets the core pathophysiological pillars of malnutrition in acute sepsis: the catastrophic loss of physiological reserve and the ensuing protein-energy wasting (34, 35). Our results suggest that in the hypercatabolic state of sepsis, the direct assessment of protein-energy balance provided by GNRI may offer a more immediate and prognostically vital readout than indices incorporating immune cells, which can be profoundly and transiently altered by the septic insult itself. Future research employing head-to-head comparisons of these indices in identical cohorts is necessary to definitively establish their relative prognostic performance and optimal use cases.

Malnutrition is defined as an imbalance between energy intake and physiological requirements (36). Nutritional status significantly impacts immune and inflammatory responses. Patients with low GNRI scores typically exhibit protein-energy malnutrition, which compromises immune defenses while promoting the release of pro-inflammatory cytokines (e.g., IL-6, TNF-α), leading to a chronic low-grade inflammatory state (37, 38). A low GNRI signifies protein-energy malnutrition, which directly compromises innate and adaptive immune function, impairing bacterial clearance and increasing susceptibility to secondary infections (39). Concurrently, malnutrition potentiates a state of chronic inflammation, characterized by elevated pro-inflammatory cytokines (e.g., IL-6, TNF-α). In sepsis, this cycle is explosively accelerated. Hypoalbuminaemia reduces endotoxin-scavenging capacity and worsens endothelial dysfunction, while the loss of muscle mass, a key endocrine organ, diminishes the production of anti-inflammatory cytokine (40–43). This confluence of immunosuppression and unabated inflammation creates a perfect storm, predisposing patients to multi-organ dysfunction and death, a trajectory directly reflected in the mortality risk we observed.

In summary, our study demonstrates across two cohorts that the GNRI—a composite marker integrating nutritional status and inflammatory levels—has significant prognostic value in critically ill patients with sepsis-associated pneumonia. Several limitations should be acknowledged. First, its observational design can establish association but not causality. Second, while we employed multiple imputation for variables with limited missing data, our initial exclusion of variables with >30% missingness, though methodologically common, could introduce selection bias if the missingness was not random and was related to both the excluded variable and the outcome. Third, our reliance on a single GNRI measurement from the first 24 h of ICU admission may not represent pre-illness nutritional status and does not capture the dynamic trajectory of nutritional risk during the ICU stay, which may hold further prognostic information. Finally, external validation in broader, more geographically diverse populations is needed to confirm the generalizability of our nomogram.

## Conclusion

Our study robustly establishes GNRI as a simple, readily available, and powerful prognostic marker in sepsis-associated pneumonia. It highlights the paramount importance of assessing nutritional risk early in the ICU course. By providing a practical tool for risk stratification, our findings pave the way for future research to investigate whether targeted nutritional interventions guided by such assessment can ultimately improve survival in this vulnerable population.

## Data Availability

Publicly available datasets were analyzed in this study. This data can be found here: https://physionet.org/content/mimiciv/3.1/.
