# Supplementary material for: Association between the geriatric nutritional risk index and 28-day mortality in critically ill sepsis-associated pneumonia patients: retrospective study based on two cohorts
Source: Front Nutr. 2026 Jan 26;12:1698973. doi: 10.3389/fnut.2025.1698973 (PMC12884061; doi:10.3389/fnut.2025.1698973)
Supplement: Supplementary file 1 [file Data_Sheet_1.docx]

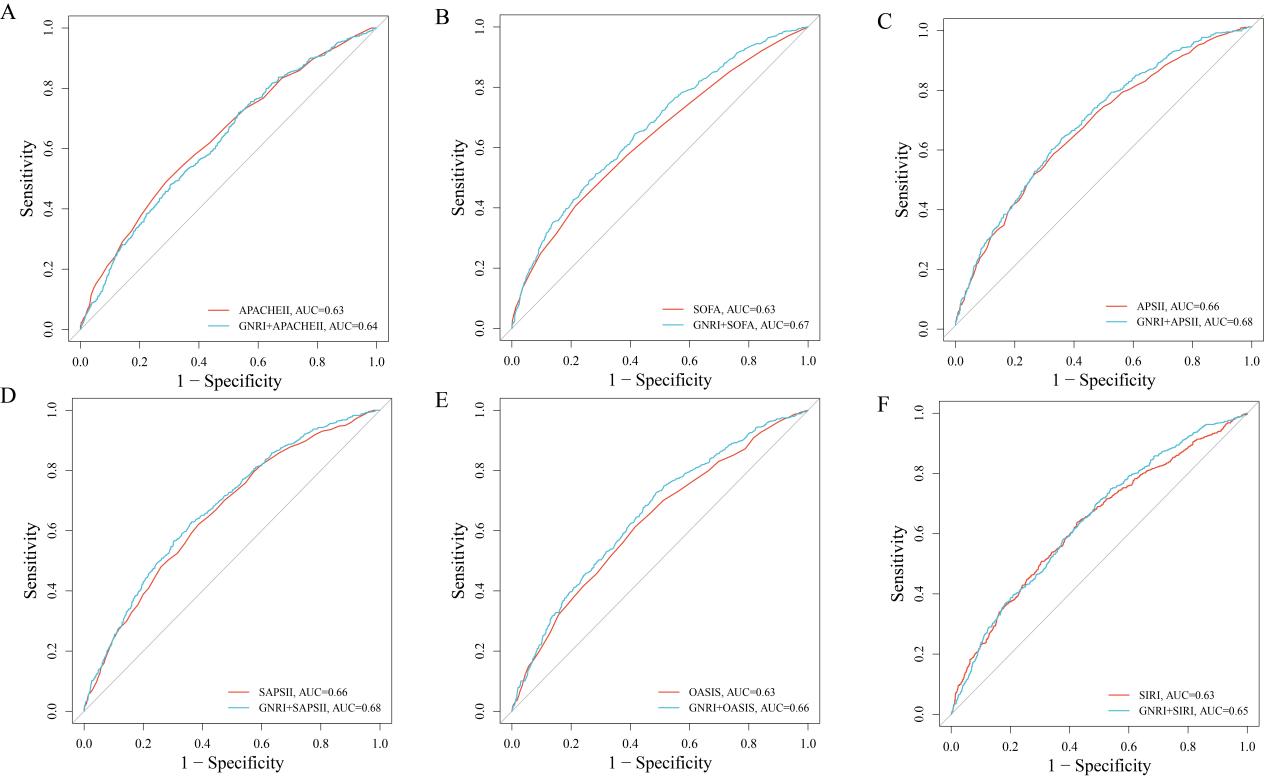


**Figure S1:** The efficacy of GNRI score in most traditional disease severity scoring models for 28 day ICU intensive care sepsis death pneumonia patients.Note: A: GNRI+APACHE II; B: GNRI+SOFA; C:GNRI+APS II; D: GNRI+SAPS II; E: GNRI+OASIS; E:GNRI+SI.


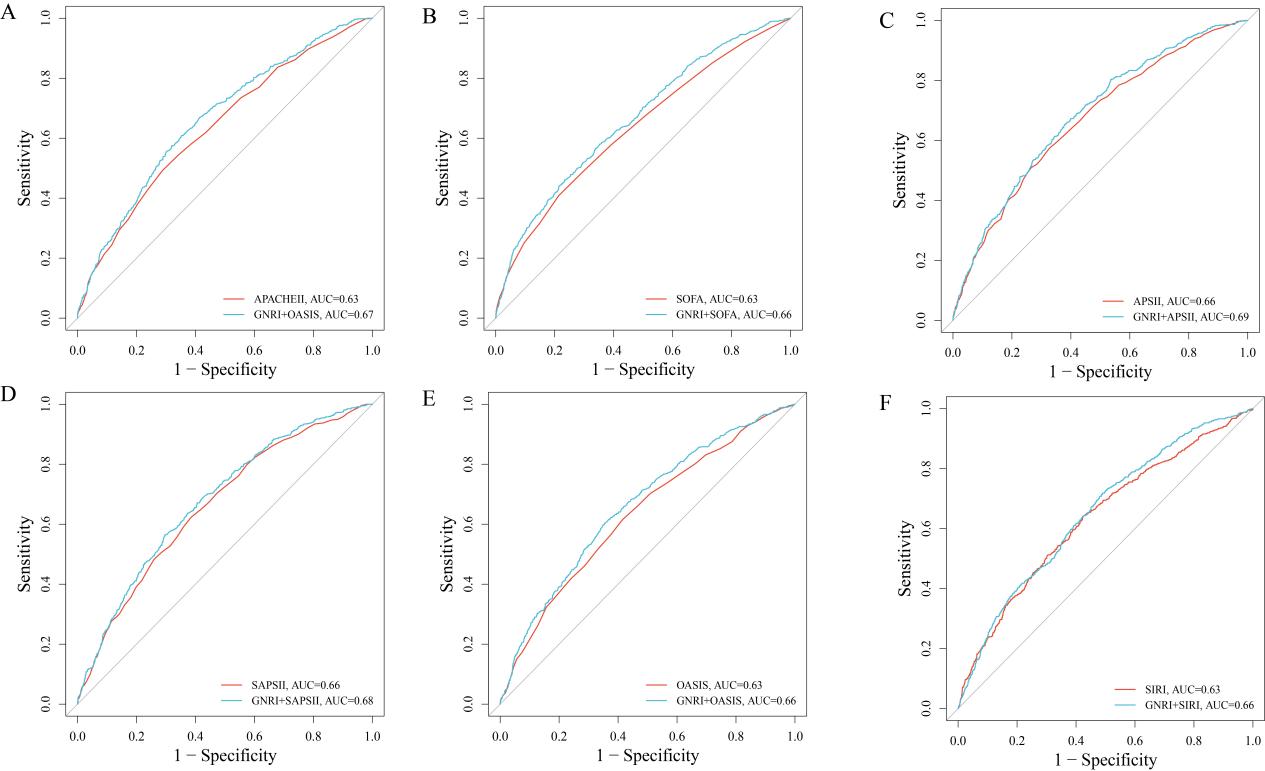


**Figure S2:** The efficacy of GNRI score in most traditional disease severity scoring models for 28 day inpatient intensive care sepsis death pneumonia patients.Note: A: GNRI+APACHE II; B: GNRI+SOFA; C:GNRI+APS II; D: GNRI+SAPS II; E: GNRI+OASIS; E:GNRI+SI.


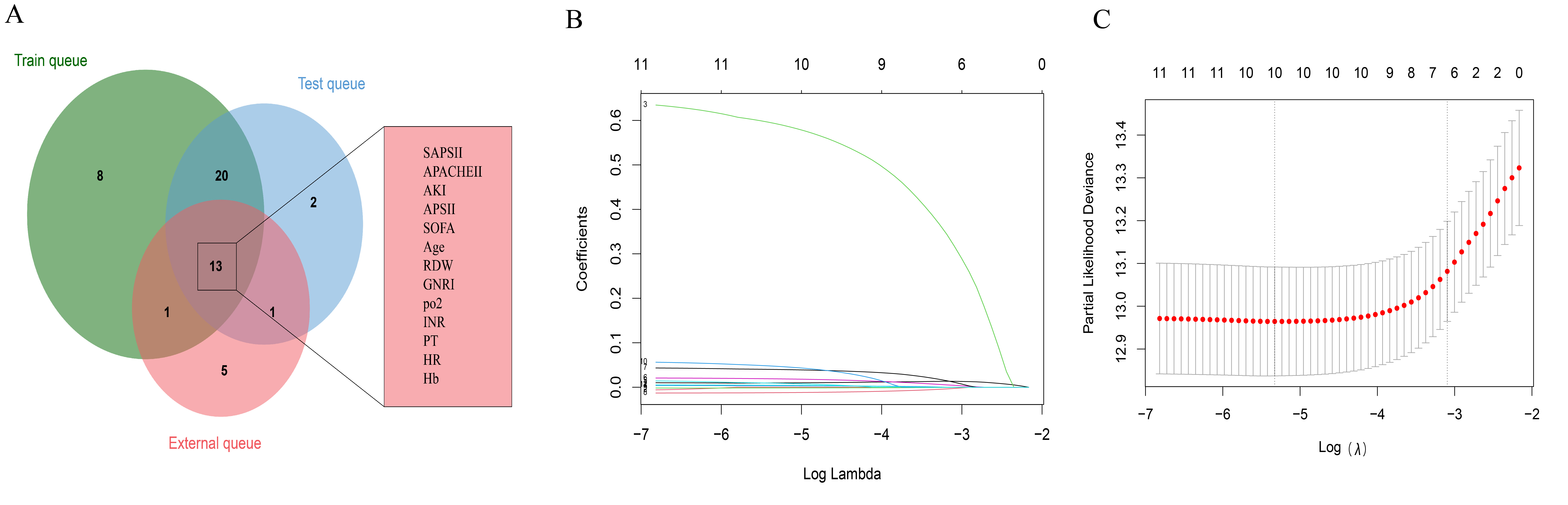


**Figure S3:** The convergence process of Veen plot and Lasso regression on candidate variables for three cohort core variables.


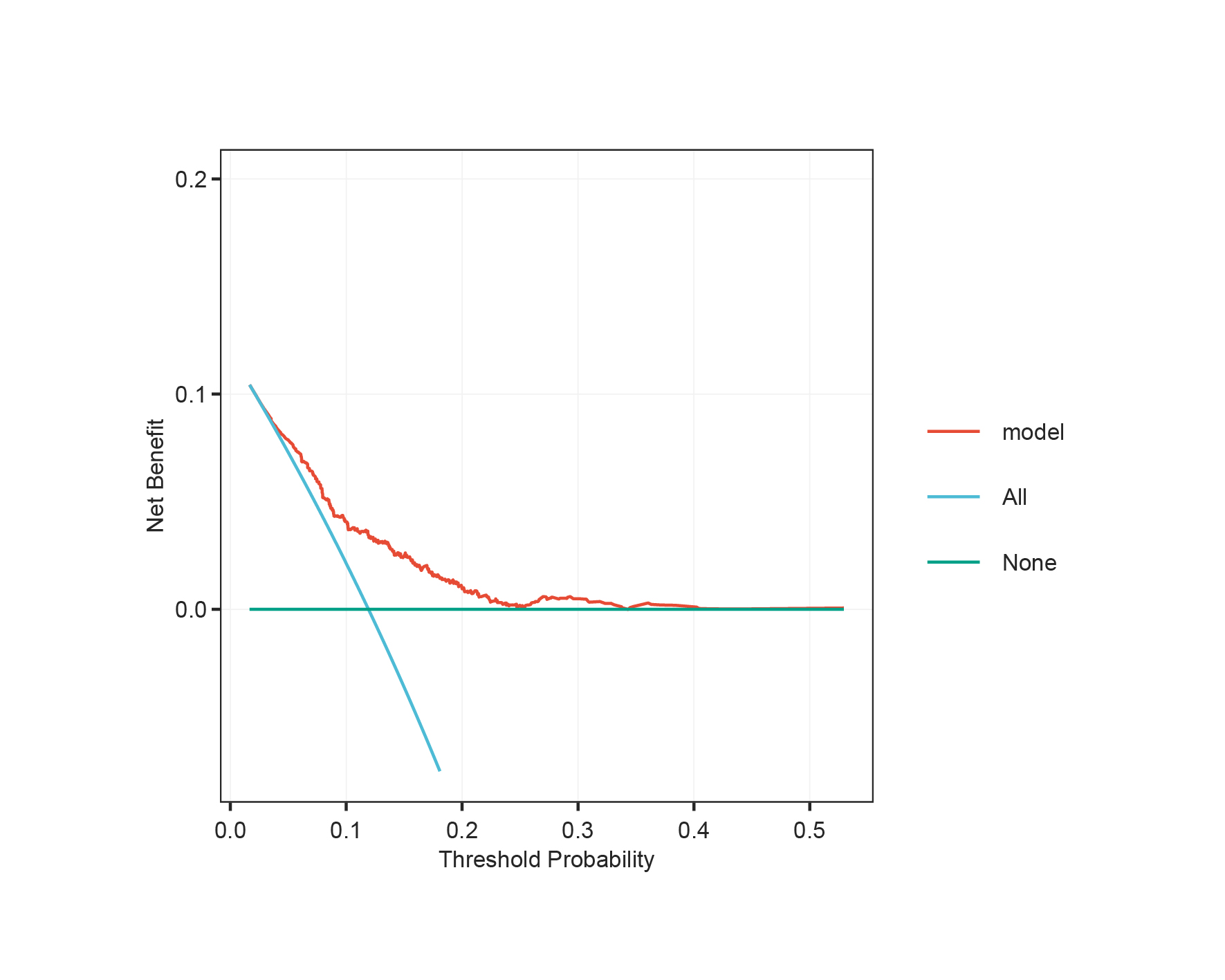


**Figure S4:** Decision curve analysis (DCA) evaluating the clinical utility of the nomogram.

Note: The decision curve compares the net benefit of using the novel nomogram for predicting 28-day mortality against two default strategies: "Treat All" (assuming all patients will die) and "Treat None" (assuming no patients will die). The net benefit is calculated across a range of threshold probabilities (the probability at which a clinician would opt for a specific intervention).


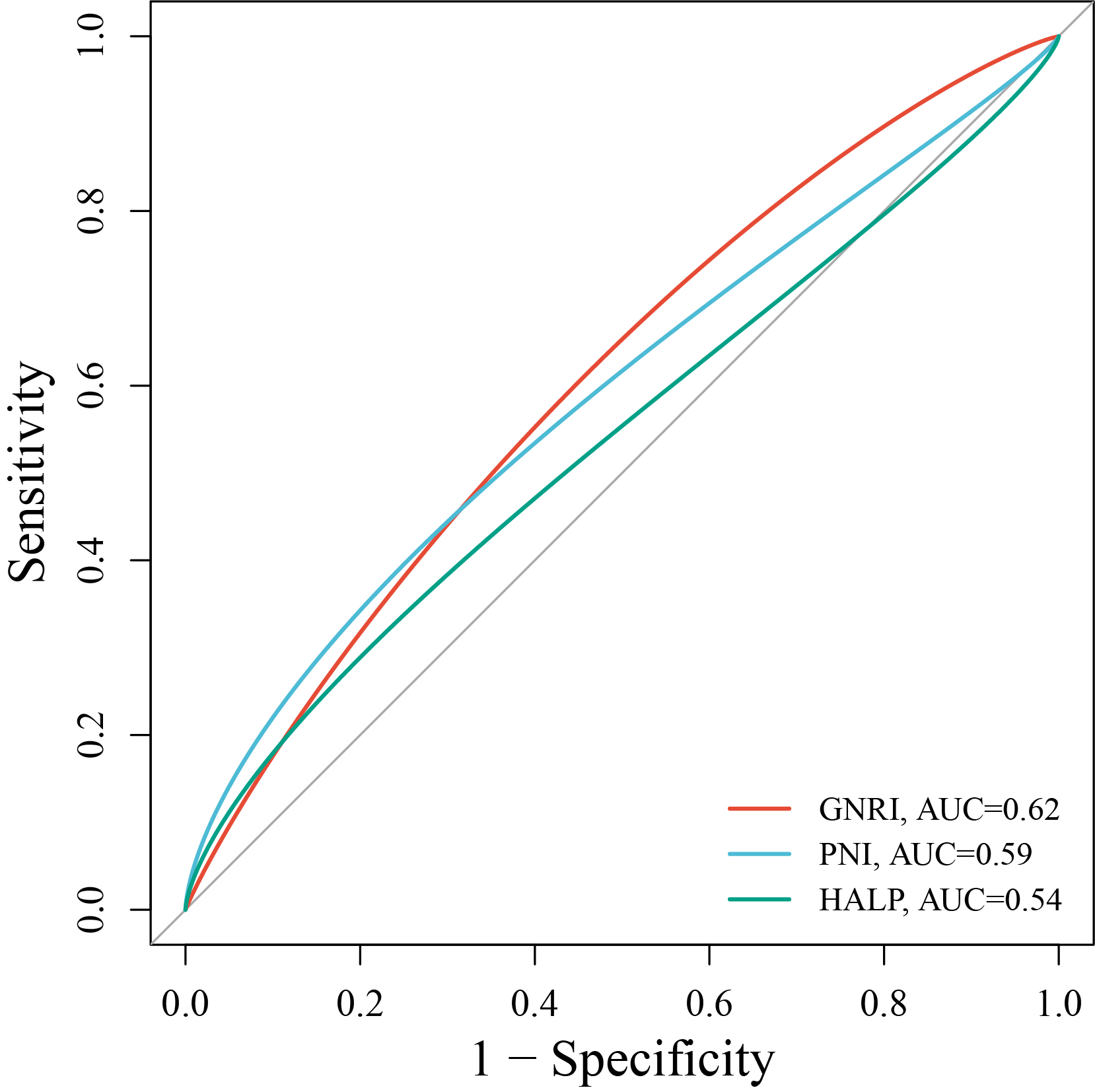


**Figure S5:** Receiver operating characteristic (ROC) curve comparison of nutritional indices for predicting 28-day mortality.

**Table S1: Summary descriptives table by groups of ICU Dead**

|  | **ALL** | **Survivor** | **No survivor** | **P overall** |
| --- | --- | --- | --- | --- |
| **Variable** | ***N=2230*** | ***N=1636*** | ***N=594*** |  |
| Gender | 1317 (59.1%) | 967 (59.1%) | 350 (58.9%) | 0.976 |
| Age | 67.3 (15.8) | 66.5 (16.4) | 69.5 (14.1) | <0.001 |
| BMI | 28.9 (8.34) | 28.8 (8.26) | 29.0 (8.56) | 0.661 |
| Race: | 1221 (54.8%) | 914 (55.9%) | 307 (51.7%) | 0.088 |
| HYPT: | 636 (28.5%) | 463 (28.3%) | 173 (29.1%) | 0.743 |
| AKI: | 1447 (64.9%) | 972 (59.4%) | 475 (80.0%) | <0.001 |
| LIC: | 274 (12.3%) | 160 (9.78%) | 114 (19.2%) | <0.001 |
| Cva: | 170 (7.62%) | 134 (8.19%) | 36 (6.06%) | 0.113 |
| CKD: | 652 (29.2%) | 490 (30.0%) | 162 (27.3%) | 0.239 |
| T2dm: | 708 (31.7%) | 545 (33.3%) | 163 (27.4%) | 0.010 |
| T1dm: | 47 (2.11%) | 41 (2.51%) | 6 (1.01%) | 0.045 |
| Hld: | 855 (38.3%) | 650 (39.7%) | 205 (34.5%) | 0.028 |
| Cb: | 448 (20.1%) | 320 (19.6%) | 128 (21.5%) | 0.329 |
| HF: | 930 (41.7%) | 711 (43.5%) | 219 (36.9%) | 0.006 |
| IHD: | 906 (40.6%) | 687 (42.0%) | 219 (36.9%) | 0.033 |
| COPD: | 521 (23.4%) | 375 (22.9%) | 146 (24.6%) | 0.447 |
| COV: | 65 (2.91%) | 31 (1.89%) | 34 (5.72%) | <0.001 |
| VP: | 2053 (92.1%) | 1508 (92.2%) | 545 (91.8%) | 0.811 |
| CRRT: | 288 (12.9%) | 135 (8.25%) | 153 (25.8%) | <0.001 |
| SOFA | 7.42 (3.60) | 6.93 (3.30) | 8.78 (4.02) | <0.001 |
| APSII | 56.8 (22.3) | 53.3 (20.2) | 66.6 (24.7) | <0.001 |
| SAPSII | 44.2 (14.3) | 42.1 (13.5) | 50.1 (14.7) | <0.001 |
| OASIS | 36.1 (8.58) | 35.1 (8.29) | 39.1 (8.68) | <0.001 |
| CCI | 6.21 (3.22) | 6.03 (3.24) | 6.71 (3.13) | <0.001 |
| PESI | 130 (27.3) | 128 (27.9) | 137 (24.3) | <0.001 |
| SI | 1.35 (0.70) | 1.28 (0.62) | 1.56 (0.84) | <0.001 |
| APACHEII | 21.9 (7.46) | 21.0 (7.06) | 24.7 (7.82) | <0.001 |
| Lym | 0.94 (0.55;1.48) | 0.99 (0.61;1.53) | 0.80 (0.43;1.32) | <0.001 |
| HCT | 32.2 (7.36) | 32.5 (7.26) | 31.4 (7.57) | 0.001 |
| Hb | 10.3 (2.44) | 10.4 (2.43) | 10.0 (2.44) | 0.003 |
| PLT | 185 (123;255) | 191 (131;262) | 158 (100;236) | <0.001 |
| RDW | 16.0 (2.77) | 15.8 (2.64) | 16.6 (3.05) | <0.001 |
| Red | 3.49 (0.86) | 3.55 (0.85) | 3.35 (0.87) | <0.001 |
| WBC | 12.3 (8.20;17.7) | 12.1 (8.20;17.2) | 12.9 (8.40;19.4) | 0.025 |
| Neu | 9.95 (6.24;14.9) | 9.56 (6.08;14.4) | 11.2 (7.04;17.3) | <0.001 |
| ALB | 2.91 (0.59) | 2.98 (0.58) | 2.74 (0.59) | <0.001 |
| AG | 15.6 (5.04) | 15.5 (4.93) | 16.1 (5.31) | 0.013 |
| Ca | 8.31 (0.86) | 8.35 (0.85) | 8.20 (0.90) | <0.001 |
| Cl | 102 (7.46) | 102 (7.24) | 102 (8.03) | 0.315 |
| Potassium | 4.32 (0.83) | 4.32 (0.81) | 4.31 (0.88) | 0.815 |
| Sodium | 138 (6.27) | 138 (5.97) | 138 (7.05) | 0.963 |
| TCO2 | 24.7 (6.21) | 24.9 (6.15) | 24.0 (6.32) | 0.002 |
| Lac | 1.80 (1.30;2.90) | 1.70 (1.20;2.70) | 2.10 (1.40;3.38) | <0.001 |
| po2 | 63.0 (42.0;105) | 65.0 (42.0;109) | 61.0 (42.0;97.8) | 0.318 |
| INR | 1.30 (1.20;1.70) | 1.30 (1.20;1.60) | 1.40 (1.20;1.90) | <0.001 |
| PT | 14.7 (12.8;18.5) | 14.4 (12.6;17.5) | 15.6 (13.2;21.2) | <0.001 |
| APTT | 31.5 (27.7;39.1) | 31.1 (27.6;37.8) | 33.0 (28.1;44.0) | <0.001 |
| TG | 148 (98.0;235) | 148 (97.8;231) | 150 (99.0;252) | 0.126 |
| ALT | 26.0 (15.0;58.0) | 25.0 (14.0;52.0) | 31.0 (17.0;69.8) | <0.001 |
| AST | 40.0 (23.0;95.0) | 37.0 (22.0;83.2) | 50.0 (27.0;128) | <0.001 |
| TB | 0.60 (0.40;1.30) | 0.60 (0.40;1.10) | 0.80 (0.40;1.80) | <0.001 |
| CRE | 1.30 (0.90;2.00) | 1.20 (0.80;2.00) | 1.40 (0.90;2.20) | 0.001 |
| UREA | 27.0 (16.0;44.0) | 25.0 (15.0;42.0) | 31.0 (20.0;49.8) | <0.001 |
| UC | 6.30 (4.60;8.30) | 6.10 (4.50;8.00) | 6.85 (4.70;9.10) | <0.001 |
| LDH | 325 (241;485) | 306 (231;449) | 382 (270;619) | <0.001 |
| UC | 6.53 (2.74) | 6.37 (2.62) | 6.98 (3.01) | <0.001 |
| LDH | 555 (1056) | 482 (783) | 757 (1564) | <0.001 |
| HR | 93.5 (21.8) | 92.4 (21.9) | 96.6 (21.4) | <0.001 |
| NBPS | 120 (24.6) | 121 (24.6) | 117 (24.5) | 0.006 |
| NBPD | 70.2 (18.7) | 70.7 (18.9) | 68.9 (17.9) | 0.033 |
| NBPM | 83.3 (18.7) | 83.9 (19.0) | 81.7 (17.9) | 0.012 |
| RR | 21.6 (7.15) | 21.3 (7.12) | 22.3 (7.19) | 0.005 |
| Spo2 | 95.8 (4.74) | 96.0 (4.62) | 95.3 (5.04) | 0.005 |
| GNRI | 84.3 (9.24) | 85.3 (9.12) | 81.7 (9.06) | <0.001 |
| GNRI group: |  |  |  | <0.001 |
| High | 915 (41.0%) | 603 (36.9%) | 312 (52.5%) |  |
| Moderate | 827 (37.1%) | 622 (38.0%) | 205 (34.5%) |  |
| Low | 337 (15.1%) | 279 (17.1%) | 58 (9.76%) |  |
| No | 151 (6.77%) | 132 (8.07%) | 19 (3.20%) |  |

**Table S2: Summary descriptives table by groups of Hosp Dead**

|  | **ALL** | **Survivor** | **No survivor** | **P overall** |
| --- | --- | --- | --- | --- |
| **Variable** | ***N=2230*** | ***N=1637*** | ***N=593*** |  |
| Gender | 1317 (59.1%) | 967 (59.1%) | 350 (59.0%) | 1.000 |
| Age | 67.3 (15.8) | 66.5 (16.4) | 69.5 (14.1) | <0.001 |
| BMI | 28.9 (8.34) | 28.9 (8.26) | 29.0 (8.57) | 0.685 |
| Race: | 1221 (54.8%) | 915 (55.9%) | 306 (51.6%) | 0.080 |
| HYPT: | 636 (28.5%) | 465 (28.4%) | 171 (28.8%) | 0.884 |
| AKI: | 1447 (64.9%) | 974 (59.5%) | 473 (79.8%) | <0.001 |
| LIC: | 274 (12.3%) | 160 (9.77%) | 114 (19.2%) | <0.001 |
| Cva: | 170 (7.62%) | 135 (8.25%) | 35 (5.90%) | 0.080 |
| CKD: | 652 (29.2%) | 489 (29.9%) | 163 (27.5%) | 0.298 |
| T2dm: | 708 (31.7%) | 544 (33.2%) | 164 (27.7%) | 0.014 |
| T1dm: | 47 (2.11%) | 41 (2.50%) | 6 (1.01%) | 0.045 |
| Hld: | 855 (38.3%) | 650 (39.7%) | 205 (34.6%) | 0.031 |
| Cb: | 448 (20.1%) | 319 (19.5%) | 129 (21.8%) | 0.262 |
| HF: | 930 (41.7%) | 710 (43.4%) | 220 (37.1%) | 0.009 |
| IHD: | 906 (40.6%) | 687 (42.0%) | 219 (36.9%) | 0.037 |
| COPD: | 521 (23.4%) | 374 (22.8%) | 147 (24.8%) | 0.367 |
| COV: | 65 (2.91%) | 31 (1.89%) | 34 (5.73%) | <0.001 |
| VP: | 2053 (92.1%) | 1509 (92.2%) | 544 (91.7%) | 0.800 |
| CRRT: | 288 (12.9%) | 135 (8.25%) | 153 (25.8%) | <0.001 |
| SOFA | 7.42 (3.60) | 6.93 (3.29) | 8.80 (4.03) | <0.001 |
| APSII | 56.8 (22.3) | 53.2 (20.2) | 66.7 (24.7) | <0.001 |
| SAPSII | 44.2 (14.3) | 42.0 (13.5) | 50.1 (14.7) | <0.001 |
| OASIS | 36.1 (8.58) | 35.1 (8.29) | 39.1 (8.67) | <0.001 |
| CCI | 6.21 (3.22) | 6.02 (3.24) | 6.74 (3.14) | <0.001 |
| PESI | 130 (27.3) | 128 (27.9) | 137 (24.3) | <0.001 |
| SI | 1.35 (0.70) | 1.28 (0.62) | 1.57 (0.84) | <0.001 |
| APACHEII | 21.9 (7.46) | 20.9 (7.05) | 24.7 (7.83) | <0.001 |
| Lym | 0.94 (0.55;1.48) | 0.98 (0.61;1.52) | 0.80 (0.43;1.32) | <0.001 |
| HCT | 32.2 (7.36) | 32.5 (7.26) | 31.4 (7.57) | 0.001 |
| Hb | 10.3 (2.44) | 10.4 (2.43) | 10.0 (2.44) | 0.003 |
| PLT | 185 (123;255) | 191 (131;262) | 157 (100;235) | <0.001 |
| RDW | 16.0 (2.77) | 15.8 (2.64) | 16.6 (3.05) | <0.001 |
| Red | 3.49 (0.86) | 3.55 (0.85) | 3.35 (0.87) | <0.001 |
| WBC | 12.3 (8.20;17.7) | 12.2 (8.20;17.2) | 12.8 (8.40;19.3) | 0.037 |
| Neu | 9.95 (6.24;14.9) | 9.56 (6.09;14.4) | 11.1 (6.96;17.2) | <0.001 |
| ALB | 2.91 (0.59) | 2.98 (0.58) | 2.73 (0.59) | <0.001 |
| AG | 15.6 (5.04) | 15.5 (4.93) | 16.1 (5.32) | 0.011 |
| Ca | 8.31 (0.86) | 8.35 (0.85) | 8.20 (0.91) | <0.001 |
| Cl | 102 (7.46) | 102 (7.24) | 102 (8.03) | 0.359 |
| Potassium | 4.32 (0.83) | 4.32 (0.81) | 4.32 (0.88) | 0.859 |
| Sodium | 138 (6.27) | 138 (5.97) | 138 (7.05) | 0.970 |
| TCO2 | 24.7 (6.21) | 24.9 (6.15) | 24.0 (6.33) | 0.002 |
| Lac | 1.80 (1.30;2.90) | 1.70 (1.20;2.70) | 2.10 (1.40;3.40) | <0.001 |
| pco2 | 45.3 (13.1) | 45.2 (12.7) | 45.3 (14.2) | 0.964 |
| ph | 7.34 (0.11) | 7.34 (0.10) | 7.33 (0.12) | 0.009 |
| po2 | 63.0 (42.0;105) | 65.0 (42.0;109) | 61.0 (42.0;97.0) | 0.310 |
| INR | 1.30 (1.20;1.70) | 1.30 (1.20;1.60) | 1.40 (1.20;1.90) | <0.001 |
| PT | 14.7 (12.8;18.5) | 14.4 (12.6;17.5) | 15.7 (13.2;21.2) | <0.001 |
| APTT | 31.5 (27.7;39.1) | 31.1 (27.6;37.8) | 33.1 (28.1;44.1) | <0.001 |
| TG | 148 (98.0;235) | 148 (97.0;231) | 150 (99.0;252) | 0.123 |
| ALT | 26.0 (15.0;58.0) | 25.0 (14.0;52.0) | 31.0 (17.0;70.0) | <0.001 |
| AST | 40.0 (23.0;95.0) | 37.0 (22.0;83.0) | 50.0 (27.0;128) | <0.001 |
| TB | 0.60 (0.40;1.30) | 0.60 (0.40;1.10) | 0.80 (0.40;1.80) | <0.001 |
| CRE | 1.30 (0.90;2.00) | 1.20 (0.80;2.00) | 1.40 (0.90;2.20) | 0.001 |
| UREA | 27.0 (16.0;44.0) | 25.0 (15.0;42.0) | 31.0 (20.0;50.0) | <0.001 |
| UC | 6.30 (4.60;8.30) | 6.10 (4.50;8.00) | 6.80 (4.70;9.10) | <0.001 |
| LDH | 325 (241;485) | 306 (231;449) | 382 (269;614) | <0.001 |
| HR | 93.5 (21.8) | 92.4 (21.9) | 96.5 (21.4) | <0.001 |
| NBPS | 120 (24.6) | 120 (24.6) | 117 (24.6) | 0.008 |
| NBPD | 70.2 (18.7) | 70.7 (18.9) | 68.9 (18.0) | 0.034 |
| NBPM | 83.3 (18.7) | 83.9 (19.0) | 81.7 (17.9) | 0.013 |
| RR | 21.6 (7.15) | 21.3 (7.12) | 22.3 (7.19) | 0.005 |
| Spo2 | 95.8 (4.74) | 96.0 (4.62) | 95.3 (5.04) | 0.004 |
| GNRI | 84.3 (9.24) | 85.3 (9.12) | 81.7 (9.04) | <0.001 |
| GNRI group |  |  |  | <0.001 |
| High | 915 (41.0%) | 602 (36.8%) | 313 (52.8%) |  |
| Moderate | 827 (37.1%) | 623 (38.1%) | 204 (34.4%) |  |
| Low | 337 (15.1%) | 279 (17.0%) | 58 (9.78%) |  |
| No | 151 (6.77%) | 133 (8.12%) | 18 (3.04%) |  |

**Table S3: Summary descriptives table by groups of external verification cohort Dead**

|  | **ALL** | **Survive** | **No-survive** | **P overall** |
| --- | --- | --- | --- | --- |
| **Variable** | ***N=245*** | ***N=175*** | ***N=70*** |  |
| Gender: | 164 (66.9%) | 113 (64.6%) | 51 (72.9%) | 0.273 |
| Age | 69.0 (59.0;78.0) | 69.0 (58.5;78.0) | 69.5 (61.0;76.8) | 0.781 |
| BMI | 27.1 (23.9;32.9) | 27.3 (23.9;33.8) | 26.4 (23.8;31.6) | 0.275 |
| HYPT: | 64 (26.1%) | 46 (26.3%) | 18 (25.7%) | 0.978 |
| AKI: | 158 (64.5%) | 105 (60.0%) | 53 (75.7%) | 0.030 |
| Cva: | 19 (7.76%) | 12 (6.86%) | 7 (10.0%) | 0.571 |
| CKD: | 62 (25.3%) | 47 (26.9%) | 15 (21.4%) | 0.471 |
| T2dm: | 90 (36.7%) | 63 (36.0%) | 27 (38.6%) | 0.818 |
| HF: | 106 (43.3%) | 78 (44.6%) | 28 (40.0%) | 0.300 |
| COPD: | 54 (22.0%) | 32 (18.3%) | 22 (31.4%) | 0.038 |
| VP: | 212 (86.5%) | 148 (84.6%) | 64 (91.4%) | 0.225 |
| SOFA | 7.00 (5.00;10.0) | 7.00 (5.00;9.00) | 8.00 (5.00;12.0) | 0.032 |
| APSII | 53.0 (42.0;66.0) | 50.0 (40.0;62.0) | 59.5 (48.2;83.8) | <0.001 |
| SAPSII | 43.0 (34.0;52.0) | 42.0 (33.5;50.0) | 46.5 (38.0;57.0) | 0.012 |
| OASIS | 36.0 (29.0;41.0) | 35.0 (29.0;40.0) | 37.0 (31.2;42.8) | 0.069 |
| SIRI | 1.24 (0.98;1.63) | 1.19 (0.95;1.52) | 1.46 (1.12;1.88) | 0.001 |
| APACHEII | 21.0 (17.0;26.0) | 21.0 (17.0;25.0) | 24.0 (18.0;30.0) | 0.002 |
| Lym | 0.97 (0.54;1.51) | 1.17 (0.66;1.57) | 0.68 (0.42;1.22) | <0.001 |
| HCT | 32.2 (26.8;37.5) | 32.9 (26.7;38.0) | 31.7 (28.1;36.2) | 0.661 |
| Hb | 10.4 (8.50;11.9) | 10.5 (8.40;12.1) | 10.2 (8.80;11.3) | 0.569 |
| PLT | 172 (117;240) | 173 (120;244) | 162 (102;219) | 0.420 |
| RDW | 15.3 (14.1;17.2) | 15.2 (13.9;17.0) | 15.9 (14.5;17.6) | 0.078 |
| Red | 3.52 (2.92;4.08) | 3.59 (2.83;4.16) | 3.42 (3.10;3.92) | 0.384 |
| WBC | 12.1 (7.50;16.7) | 11.9 (7.60;15.9) | 12.2 (7.50;17.3) | 0.717 |
| Neu | 9.48 (5.67;14.2) | 8.84 (5.46;14.0) | 10.7 (6.91;16.6) | 0.076 |
| ALB | 3.00 (2.70;3.40) | 3.20 (2.80;3.50) | 3.00 (2.52;3.20) | 0.003 |
| AG | 15.0 (13.0;18.0) | 15.0 (13.0;18.0) | 15.0 (13.0;17.0) | 0.654 |
| Ca | 8.30 (7.90;8.80) | 8.30 (7.85;8.80) | 8.30 (8.00;8.80) | 0.869 |
| Cl | 102 (99.0;107) | 102 (98.5;106) | 103 (99.0;107) | 0.452 |
| Potassium | 4.10 (3.70;4.70) | 4.20 (3.75;4.70) | 4.10 (3.60;4.80) | 0.516 |
| Sodium | 139 (134;142) | 138 (134;141) | 140 (134;144) | 0.204 |
| TCO2 | 24.0 (21.0;28.0) | 24.0 (21.0;28.0) | 24.5 (19.2;30.5) | 0.571 |
| Lac | 1.80 (1.20;2.90) | 1.70 (1.20;2.85) | 2.05 (1.30;2.98) | 0.313 |
| pco2 | 42.0 (36.0;49.0) | 41.0 (36.0;48.5) | 43.5 (36.2;50.0) | 0.253 |
| ph | 7.35 (7.28;7.41) | 7.36 (7.29;7.41) | 7.33 (7.24;7.43) | 0.422 |
| po2 | 62.0 (43.0;111) | 67.0 (44.0;125) | 52.5 (42.2;75.8) | 0.017 |
| INR | 1.30 (1.20;1.70) | 1.30 (1.10;1.60) | 1.40 (1.20;1.80) | 0.107 |
| PT | 14.6 (12.5;18.1) | 14.5 (12.3;17.4) | 15.1 (12.8;19.5) | 0.084 |
| APTT | 32.1 (28.1;39.8) | 31.2 (27.7;39.7) | 34.2 (28.4;41.5) | 0.349 |
| TG | 146 (96.0;209) | 144 (88.5;186) | 168 (111;255) | 0.047 |
| ALT | 24.0 (14.0;43.0) | 24.0 (13.5;42.5) | 24.0 (15.2;48.5) | 0.548 |
| AST | 39.0 (24.0;83.0) | 39.0 (24.0;77.5) | 41.0 (30.0;86.0) | 0.344 |
| TB | 0.70 (0.40;1.50) | 0.60 (0.40;1.25) | 0.90 (0.40;1.90) | 0.050 |
| CRE | 1.20 (0.80;1.90) | 1.20 (0.80;1.90) | 1.25 (0.83;1.87) | 0.992 |
| UREA | 21.0 (13.0;37.0) | 23.0 (14.0;40.0) | 18.0 (11.2;27.8) | 0.014 |
| UC | 5.40 (4.10;7.60) | 5.80 (4.30;7.60) | 4.65 (3.52;7.30) | 0.061 |
| LDH | 284 (222;405) | 304 (234;434) | 262 (214;357) | 0.025 |
| HR | 91.0 (78.0;109) | 88.0 (77.0;105) | 97.5 (84.0;113) | 0.009 |
| NBPS | 116 (103;133) | 120 (106;134) | 112 (97.0;128) | 0.025 |
| NBPD | 66.0 (57.0;81.0) | 68.0 (57.0;82.5) | 64.0 (57.2;71.8) | 0.116 |
| NBPM | 80.0 (69.0;93.0) | 81.0 (69.0;94.5) | 77.5 (68.0;86.0) | 0.089 |
| RR | 20.0 (16.0;24.0) | 20.0 (16.0;24.0) | 19.5 (16.0;24.8) | 0.639 |
| Spo2 | 97.0 (94.0;100) | 97.0 (94.0;99.5) | 96.0 (94.0;100) | 0.897 |
| GNRI | 86.4 (81.9;92.3) | 87.9 (83.4;92.3) | 86.4 (78.9;89.3) | 0.008 |
| GNRI group: |  |  |  | 0.027 |
| High | 67 (27.3%) | 39 (22.3%) | 28 (40.0%) |  |
| Moderate | 109 (44.5%) | 83 (47.4%) | 26 (37.1%) |  |
| Low | 43 (17.6%) | 31 (17.7%) | 12 (17.1%) |  |
| No | 26 (10.6%) | 22 (12.6%) | 4 (5.71%) |  |
